# An Overdose of Gold

**Published:** 1892-06

**Authors:** 


### An Overdose of Gold.

In December last a French soldier met with death by gold instead of lead. Having been accused of the theft of a sum of money, he followed the course, not unknown in the Orient, of secreting the stolen cash in his stomach. He swallowed the money and by so doing was acquitted by the judge because there was no tangible or exterior evidence of gilt. He was set free but disease presently arrested him; he became the subject of an acute attack of indigestion due to the indigested gold. This attack subsequently resulted in death. An autopsy was performed and there was found in his stomach twenty-one gold coins of a value of eighty-five dollars to some former owner. The lethal dose of gold has not been recorded hitherto in the text-books on toxicology but it can in the future be stated that the quantity above mentioned "has been followed by a fatal result."

